# Detection of foot-and-mouth disease virus in raw milk in Menofia Governorate and its effect on reproductive hormones and physiochemical properties of milk

**DOI:** 10.14202/vetworld.2022.2202-2209

**Published:** 2022-09-16

**Authors:** Ashraf Khamees Shaban, Ragab Hassan Mohamed, Asem Mohammed Zakaria, Eman Mohamed Baheeg

**Affiliations:** 1 Shebeen El-Kome Branch, Animal Health Research Institute (AHRI), Agricultural Research Center, Dokki, Giza, Egypt; 2 Department of Theriogenology, Faculty of Veterinary Medicine, Aswan University, Aswan, Egypt; 3 Department of Food Hygiene, Faculty of Veterinary Medicine, Aswan University, Aswan, Egypt; 4 ELISA Unite and Viral Strain Bank, Animal Health Research Institute (AHRI), Agricultural Research Center, Dokki, Giza, Egypt

**Keywords:** foot-and-mouth disease, physiochemical properties of milk, raw milk, reproductive hormones

## Abstract

**Background and Aim:**

Foot-and-mouth disease (FMD) is an extremely contagious viral disease that affects domestic and wild cloven-hoofed animals. In Egypt, FMD has been enzootic since the 1950s and caused great economic losses in cattle and buffalos over the past few years. This study aimed to detect FMD virus (FMDV) in serum and raw milk samples collected from infected and adjacent cattle and buffalos from different localities in El Menofia Governorate, Egypt.

**Materials and Methods:**

Blood and milk samples were collected from apparently diseased and adjacent 100 cows and 100 buffalos. Serum samples were prepared and used for the detection of FMDV using a non-structural protein enzyme-linked immunosorbent assay, while real-time reverse transcription-polymerase chain reaction (rRT-PCR) was used for the detection of FMDV in milk samples. Reproductive hormones were estimated using radioimmunoassay kits. Milk constituents were determined by Lactoscan.

**Results:**

Of the 200 examined serum samples (100 cows and 100 buffalos), 56% and 44% were seropositive for FMDV non-structural protein antibodies in cattle and buffalo, respectively. Real-time reverse transcription-polymerase chain reaction results confirmed that all examined milk samples collected from seropositive animals were positive for FMDV. Estrogen and progesterone levels in the serum of seropositive and seronegative animals were measured, and FMDV was proven to significantly elevate estrogen and reduce progesterone levels in both non-pregnant and pregnant animals during different stages of pregnancy. The effect of the virus on milk composition and somatic cell count (SCC) was also studied, revealing that FMDV infection significantly decreased the level of milk fat, protein, and lactose but did not significantly affected minerals, pH, and conductivity. Moreover, it significantly increased the SCC.

**Conclusion:**

Data recorded in this study indicates a widespread occurrence of FMDV in cattle and buffalo all over Menofia Governorate, Egypt. Infected raw milk is of poor quality and, if put for commercial sale, may have health risks for consumers and play a significant role in spreading the virus. Moreover, FMDV may disturb some reproductive hormones, which could adversely affect cattle and buffalo productivity. Therefore, preventive programs and accurate diagnosis are essential for successful disease control.

## Introduction

Foot-and-mouth disease (FMD), the first animal pathogen identified as a virus, was initially described in the 16^th^ century [1, 2]. According to Office International des Epizooties, it is one of the most significant diseases threatening the international trade of animals and their products [1]. The first outbreak of FMD in Egypt was caused by SAT2 in 1950 and by strain A in 1952, 1956, and 1958. Several foci were detected from 1961 until 2006 with strains O and O1 until the appearance of strain A in the FMD epidemic that occurred between January and June 2006 [3, 4]. In 2012, there was a dramatic upsurge in FMD outbreaks in Egypt related to the SAT2 strain [5]. The disease is caused by FMD virus (FMDV), which is a small non-enveloped, icosahedral virus that belongs to the genus *Aphthovirus* of the family *Picornaviridae* [2]. Foot-and-mouth disease virus possesses a positive sense, single-stranded linear RNA genome of approximately 8.5 kb in length (with a poly (A) sequence at the 3’-end) that codes for 12 proteins, four of which are structural proteins (VP1–4) that make up the capsid of the virus, and eight of which are non-structural proteins (non-structural proteins [NSPs]; 2A, 3A, 2B, 3B, 2C, 3C, 3D, and L) that participate in replication of the virus and play many functions in the host cell [6]. Clinically, FMD is characterized by fever and vesicle formation in and around the mouth, feet, and mammary glands, which often rapidly rupture and become erosive lesions. Other symptoms are excessive salivation, depression, anorexia, lameness, and reluctance to move or rise. Possible complications include chronic lameness, temporary or permanent decrease in milk production, weight loss, mastitis, and loss of condition. Deaths usually occur only in young animals as a result of multifocal myocarditis [7].

Milk and milk products are very important commodities in international trade. Their quality and safety are major areas of concern for producers, consumers, and public health officials worldwide [8], and they play a significant role in the transmission of FMDV and other disease agents. Foot-and-mouth disease is a zoonosis, but its occurrence in humans is quite rare. The virus has been isolated in humans and typed (type O, followed by type C and rarely A). There is one report from 1834 of three veterinarians acquiring the disease from deliberately drinking raw milk from infected cows, but there is no report of infection from pasteurized milk [9]. The incubation period in humans is 2–6 days. Symptoms in humans are mostly mild and self-limiting, mainly consisting of fever, malaise, sore throat, vomiting, ulcerative lesions of oral tissues, and uncomfortable tingling blisters on the hands, but these symptoms should not be confused with human hand, foot, and mouth disease. This is an unrelated and usually mild viral infection that mainly affects children and is caused by different viruses, principally coxsackie A virus [10].

Due to the economic implications and public health hazards of FMD, the disease has commanded international attention for its control and prevention, which depend on early diagnosis, vaccination, and strict quarantine measures in addition to good animal care [11].

Although there is a regular program to control and eradicate FMD, it is still endemic in Egypt. This study aimed to determine the extent of the spread of FMDV in different localities of Menofia Governorate, Egypt, and to study its effects on reproductive hormones and physiochemical properties of milk.

## Materials and Methods

### Ethical approval

The study was approved by Egyptian Medical Research Ethics Committee (no. 14 – 126).

### Study period and location

This study was conducted from September 2021 to February 2022. Blood and milk samples were collected from 200 infected and adjacent animals (100 cows and 100 buffalos) from different localities all over Menofia Governorate, Egypt.

### Animals

Some animals were in different stages of pregnancy while others were non-pregnant. Pregnancy was determined by rectal palpation and serial transrectal ultrasonographic examinations performed using real-time, B-mode, diagnostic ultrasound (SonoAce R3; Samsung, Medison, South Korea) equipped with a high frequency (12 MHz) endorectal transducer. The device also had a color Doppler mode.

### Blood sampling

Two hundred blood samples (10 mL each) were collected from clinically suspected and adjacent cattle and buffalos through jugular vein puncture without anticoagulant using a Vacutainer (Shanghai Sun Trine Biotechnology Company, China) under hygienic conditions. Plain tubes were centrifuged at 1006 × *g* for 15 min to obtain a clear serum. Samples were kept frozen at −20°C until used. Serum samples were used to detect FMDV by enzyme-linked immunosorbent assay (ELISA) and determine its effect on reproductive hormones. The test kits used for estradiol were KGE014, supplied by Parameter TM (USA), while the Progesterone Enzyme Immunoassay commercial test kits (Product EA 74) were supplied by Oxford Biomedical Research (Rochester Hills, Michigan, USA). All test kits were used according to their manufacturers’ instructions.

### Milk sampling

Milk samples (500 mL each) were collected individually from hand-milked cattle and buffalos in screw-capped bottles and kept frozen until examination. Foot-and-mouth disease virus in milk was detected by real-time reverse transcription-polymerase chain reaction (rRT-PCR). Milk constituents such as lactose, fat, protein, ash, pH, conductivity, and somatic cell count (SCC) were measured electronically by Lactoscan, manufactured by Milkotronic Ltd. (Bulgaria), and followed the method of Mohamed *et al*. [12].

### Foot-and-mouth disease virus reference strain

The FMDV strain was kindly provided by the Virology Department of the Animal Health Research Institute in Dokki, Giza, Egypt.

### Foot-and-mouth disease virus NSP ELISA

ELISA was carried out in the Virology Department of the Animal Health Research Institute in Dokki, Giza, Egypt. Commercial ELISA kits were obtained from Svanova Biotech AB, Sweden) to detect antibodies against FMDV NSP serum samples following the method of Dodet and Vicari [13].

### Real-time polymerase chain reaction

The QIAamp^®^ Viral RNA Mini Kit obtained from Qiagen (Germany) was used to extract viral RNA from positively isolated samples’ supernatants following the mini spin protocol according to the manufacturer’s instructions and eluted in 50 mL of elution buffer.

### Primers and probes used for rRT-PCR

The oligonucleotide primers used for common FMDV were designed (Metabion International, Germany) to share maximum homology with all seven serotypes of FMDV (universal primers for FMDV), according to Armson *et al*. [14], as shown in Table-1.

**Table-1:** Primers and probes used in rRT-PCR for the detection of FMDV.

FMD serotype	Primer	Sequences 5’- 3’	Position
Common	Callahan 3DF	ACT GGG TTT TAC AAA CCT GTG A	3D
	Callahan 3DR	GCG AGT CCT GCC ACG GA	3D
	Callahan 3DP probe	FAM- TCC TTT GCA CGC CGT GGG AC - TAMRA	3D

rRT-PCR=Real-time reverse transcription-polymerase chain reaction, FMD=Foot-and-mouth disease, FMDV=Foot-and-mouth disease virus

### One-step RT-PCR

The real-time reaction combined with the reverse transcription in routine RT-PCR was applied in the Biotechnology Research Department of the Animal Health Research Institute in Dokki, Giza, Egypt. The real-time cycler program is shown in Table-2.

**Table-2:** Real-time cycler program.

Step	Time	Temperature
Reverse transcription	30 min	50°C
PCR initial activation step	15 min	95°C
2-step cycling:		
Denaturation	15 s	94°C
Denaturation Combined annealing/extension	60 s	60°C

Number of cycles=50

### Statistical analysis

Data were analyzed with IBM Statistical Package for the Social Sciences Statistics for Windows, Version 20.0. (IBM Corp., Armonk, NY, USA). Data were entered as numerical or categorical, as appropriate. Quantitative data were presented as mean ± standard deviation. An independent sample t-test was used to measure the significance between two parametrically distributed quantitative variables. p < 0.05 was considered statistically significant.

## Results

**Figure-1:**
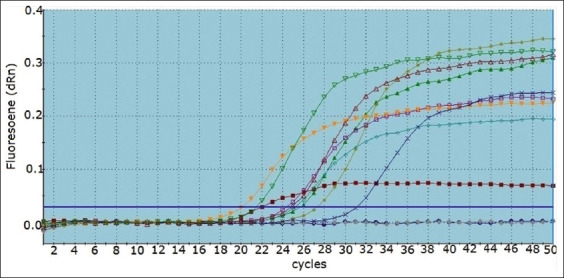
Amplification curves of sensitivity test of common foot-and-mouth disease virus using real-time polymerase chain reaction that shows positive results for examined samples and negative control sample. Positive computed tomography (CT) values ranged from 30 to 35 cycle and negative CT value considered 40 cycles.

According to the results shown in Table-3, 56% and 44% of the cattle and buffalo samples were seropositive, respectively. Real-time reverse transcription-polymerase chain reactions was used to detect FMDV in milk samples, and all milk samples collected from seropositive animals were FMD positive, as shown in Figure-1. The effects of FMDV on milk composition during different stages of pregnancy and in non-pregnant cattle and buffalo are reported in Tables-4–6, showing that milk components differed according to stage of pregnancy and that FMDV had a significant effect (p *<* 0.05) on the reduction of milk fat, protein, and lactose levels, while SCCs were increased. Minerals, pH, and conductivity were not significantly affected (p > 0.05).

**Table-3:** Results of Svanovir FMD-ELISA for the detection of FMDV non-structural protein antibodies in collected serum samples.

Animals	No. of examined samples	No. of+ve samples	% of+ve samples
Cattle	100	56	56
Buffalo	100	44	44

FMD-ELISA=Foot-and-mouth disease-enzyme-linked immunosorbent assay, FMDV=Foot-and-mouth disease virus

**Figure-2:**
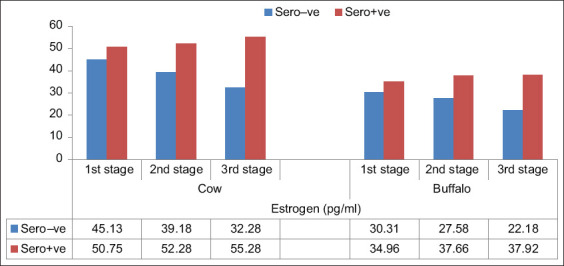
Effect of foot-and-mouth disease virus on estrogen level in cow and buffalo during different stages of pregnancy that showing elevation of estrogen level in seropositive animals.

**Figure-3:**
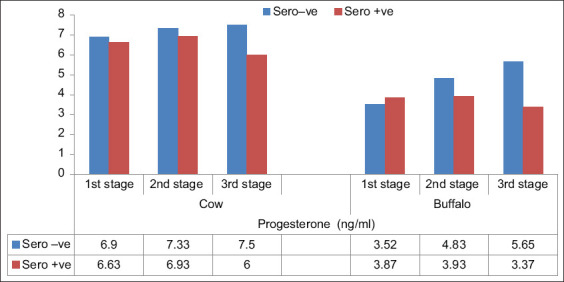
Effect of foot-and-mouth disease virus on progesterone level in cow and buffalo during different stages of pregnancy that showing reduction of progesterone level in seropositive animals.

**Figure-4:**
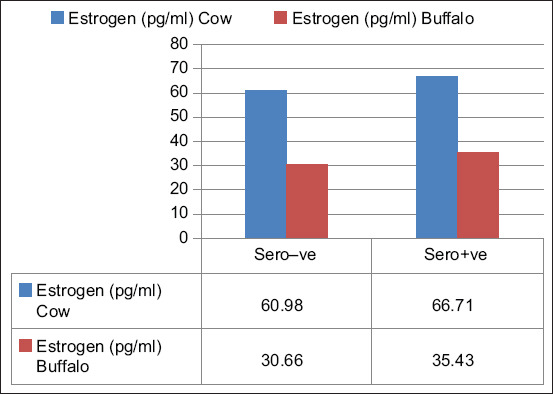
Effect of foot-and-mouth disease virus on estrogen level of non-pregnant cow and buffalo that showing elevation of estrogen level in seropositive animals.

**Figure-5:**
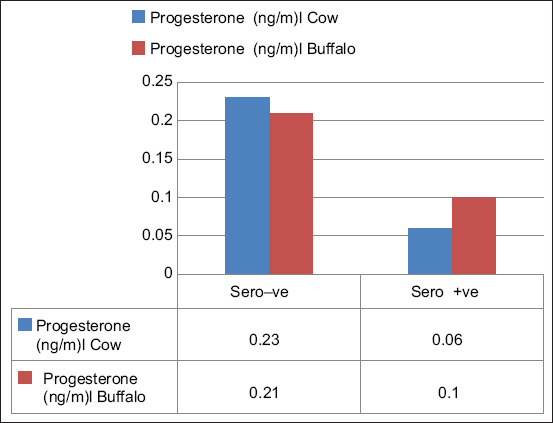
Effect of foot-and-mouth disease virus on progesterone level of non-pregnant cow and buffalo that showing reduction of progesterone level in seropositive animals.

The effect of the FMDV on serum estrogen and progesterone levels in cattle and buffalos was also studied, and the results reported in Figures-2–5 show that it had a significant effect (p *<* 0.05) on the elevation of estrogen and reduction of progesterone levels in non-pregnant and pregnant animals during different stages of pregnancy. The mean estrogen levels of seronegative cows in the first, second, and third stages of pregnancy were 45.13 ± 0.23, 39.18 ± 1.68, and 32.28 ± 1.35 pq/mL, respectively, and 50.75 ± 0.23, 52.28 ± 0.35, and 55.28 ± 0.28 pq/mL in seropositive cows, respectively. Meanwhile, the mean estrogen levels in seronegative buffalos were 30.31 ± 0.67, 27.58 ± 0.60, and 22.18 ± 1.20 pq/mL, respectively, and 34.96 ± 0.17, 37.66 ± 0.69, and 37.92 ± 0.58 pq/mL, respectively, in seropositive buffalos (Figure-2). The mean progesterone levels in seronegative cows during the first, second, and third stages of pregnancy were 6.90 ± 0.13, 7.33 ± 0.10, and 7.50 ± 0.03 ng/mL, respectively, and 6.63 ± 0.20, 6.93 ± 0.08, and 6.00 ± 0.10 ng/mL, respectively, in the seropositive cows (Figure-3). Meanwhile, in seronegative buffalos, they were 3.52 ± 0.15, 4.83 ± 0.15, and 5.65 ± 0.45 ng/mL, respectively, and 3.87 ± 0.14, 3.93 ± 0.08, and 3.37 ± 0.13 ng/mL, respectively, in seropositive buffaloes. The mean estrogen levels were 60.98 ± 0.32 and 30.66 ± 0.35 pq/mL in seronegative cows and buffalos, respectively, and 66.71 ± 1.12 and 35.43 ± 0.34 pq/mL in seropositive cows and buffalos, respectively (Figure-4). The mean progesterone levels were 0.23 ± 0.03 and 0.21 ± 0.04 ng/mL in seronegative cows and buffalos, respectively, and 0.06 ± 0.02 and 0.10 ± 0.01 ng/mL in seropositive cows and buffalos, respectively (Figure-5).

## Discussion

Foot-and-mouth disease is one of the most clinically significant viral diseases affecting cattle, buffalos, and other cloven-hoofed animals [2]. It causes severe economic losses due to its high morbidity, decreases meat and milk yields, and leads to other secondary complications [15]. Therefore, early detection and serotyping of the virus using a sensitive and rapid method for diagnosis is essential for disease control, which can be carried out through an appropriate emergency vaccine, and to help track the origin and spread of an outbreak [11]. In this study, Svanovir FMD-ELISA was used to detect FMD NSP antibodies in cattle and buffalos to differentiate between vaccinated and infected animals. The data reported in Table-3 show that of 100 cattle and 100 buffalo serum samples examined, 56 and 44 samples were found to be infected and had antibodies against the NSP of FMDV, respectively. These findings indicate previous or current viral replication among seropositive cattle and buffalo. Moreover, NSPs, unlike structural proteins, are highly conserved and, therefore, are not serotype specific, so the detection of these antibodies is not serotype restricted. The high prevalence of FMD in the study can be attributed to a variety of factors: The purchase of new animals without following quarantine protocols, communal sharing of water and feed, some farmers’ refusal to vaccinate animals or vaccine administration by non-official livestock personnel, the fact that animal farms are close to each other, indirect transmissions of FMD, and inadequate biosecurity practices.

Foot-and-mouth disease diagnosis in the field is dependent on clinical recognition followed by confirmation by objective tests that are usually carried out in specialized laboratories. During FMD viral replication in cattle and buffalos, different populations of antibodies are elicited and directed against the NSPs 2A, 2B, 2C, 3AB, 3C, and 3D, regardless of whether the animal exhibits symptoms of the disease or not [16]. Detection of antibodies to structural proteins alone is not suitable to differentiate between infected and vaccinated animals. Differentiating between these two categories of animals is important during serological surveys to detect evidence of infection in vaccinated animals [17]. Lower results of FMD detection were reported by El-Rhman *et al*. [11] and a nearly similar result was reported by Salem *et al*. [18], while higher results were reported by Ibrahim1 *et al*. [19] and Zeedan *et al*. [20] who used FMD-3ABC to distinguish FMDV-infected or carrier animals from vaccinated animals in Egypt.

Foot-and-mouth disease virus-infected animals usually shed the virus in their milk several days before clinical signs appear, so screening raw milk for FMDV can likely detect its presence sooner than farmers reporting clinical signs. Thus, this method should be suitable for national, regional, and global FMD surveillance. Moreover, milk is considered a good medium for laboratory diagnosis and surveillance of FMD in dairy herds, as it is available in plentiful quantities and can easily be collected [21]. Recently, rRT-PCR assays were developed that can detect FMDV in a variety of sample matrices. These assays have high specificity and sensitivity [22]. In this study, rRT-PCR was used to detect FMDV in raw milk samples collected from clinically infected and adjacent cattle and buffalos using oligonucleotide universal primers and probe to amplify highly conserved sequences among all seven FMDV serotypes. The result confirmed that all examined seropositive animals had FMDV in their milk, with a cycle threshold value ranged from 20 to 35 cycles (Figure-1). This result is in accordance with those of Das *et al*. [21], Ahmed *et al*. [23], and Mohamed *et al*. [24], who used RT-PCR assays for FMD diagnosis with high diagnostic and analytical sensitivity. The FMDV replicates in the mammary gland after infection, and the shed virus is incorporated into fat droplets and casein micelles within 2 days before the appearance of clinical signs, fat droplets and casein micelles aid the virus to be protected from environmental inactivants [24]. Foot-and-mouth disease virus can persist in milk and dairy products for a long time at refrigeration temperatures; it can survive in raw milk for 6 days at 18°C and for 15 days at 4°C [25]. The temperature-time conditions used for pasteurization in the dairy industry are carefully chosen to ensure milk safety but retain the quality and flavor of the milk [26]. The double set pasteurization (72°C for 30 s) and UHT (135°C for 1 s) are proven and reliable methods for inactivating the virus in raw milk [27]. In addition to the danger of FMDV transmission through milk, the virus can significantly reduce milk yields and animal productivity. This negative effect of FMDV on milk yield has been reported in many previous studies, which confirmed that the decrease in milk production began before the detection of the clinical signs and occurrence of an outbreak, with recovery usually taking 2 months after the end of the outbreak [9, 28, 29]. In general, and to the best of our knowledge, the impact of FMDV on milk composition and SCC has not yet been sufficiently studied. Therefore, in this study, milk samples were collected from both non-pregnant and pregnant seropositive and seronegative cattle and buffalos to measure the levels of different milk constituents and SCCs. The results recorded in Tables-4–6 show that FMD infection significantly reduced (p *<* 0.05) milk fat, protein, and lactose levels and increased SCC, while minerals, pH, and conductivity were not significantly affected. Foot-and-mouth disease causes fever, anorexia, ulcers, vesicles, and erosions on lips, dental pad, tongue, and gum, leading to food avoidance due to pain [29]. It may also cause mastitis and subclinical mastitis in dairy animals [30], and all these symptoms may be the cause of milk yield reduction and defects in milk components. Nearly identical results to ours were reported by Ansari-Lari *et al*. [31], who found that FMDV decreases the level of fat and protein in cow’s milk.

**Table-4:** Effect of FMD virus on cow milk composition during different stages of pregnancy.

Milk parameters	Stage of pregnancy								

	1^st^ stage			2^nd^ stage			3^rd^ stage		
		
	Sero –ve (n = 8)	Sero+ve (n = 8)	p-value	Sero –ve (n = 8)	Sero+ve (n = 8)	p-value	Sero –ve (n = 8)	Sero+ve (n = 8)	p-value
Fat	3.42 ± 0.31	3.13 ± 0.24	0.002	3.53 ± 0.27	3.18 ± 0.21	0.0001	3.61 ± 0.29	3.21 ± 0.19	0.0001
Protein	3.47 ± 0.23	3.13 ± 0.18	0.0001	3.32 ± 0.19	2.94 ± 0.16	0.0001	3.25 ± 0.21	2.87 ± 0.17	0.0001
Lactose	4.82 ± 0.14	4.39 ± 0.13	0.0001	4.65 ± 0.13	4.32 ± 0.16	0.0001	4.74 ± 0.16	4.36 ± 0.12	0.0001
Minerals	0.72 ± 0.07	0.71 ± 0.06	0.059	0.74 ± 0.05	0.72 ± 0.07	0.305	0.74 ± 0.070	0.73 ± 0.09	0.697
pH	6.57 ± 0.12	6.59 ± 0.10	0.570	6.59 ± 0.09	6.60 ± 0.06	0.681	6.61 ± 0.11	6.58 ± 0.08	0.330
Conductivity	4.19 ± 0.03	4.18 ± 0.06	0.509	4.20 ± 0.08	4.19 ± 0.04	0.620	4.21 ± 0.05	4.20 ± 0.06	0.570
SCC (×10^3^)	149.46 ± 7.4	175.54 ± 6.3	0.0001	152.35 ± 4.6	179.64 ± 5.2	0.0001	146.73 ± 5.2	183.14 ± 4.9	0.0001

FMD=Foot-and-mouth disease, SCC=Somatic cell count

**Table-5:** Effect of FMD virus on buffalo milk composition during different stages of pregnancy.

Milk parameters	Stage of pregnancy								

	1^st^ stage			2^nd^ stage			3^rd^ stage		
		
	Sero –ve (n = 8)	Sero+ve (n = 8)	p-value	Sero –ve (n = 8)	Sero+ve (n = 8)	p-value	Sero –ve (n = 8)	Sero+ve (n = 8)	p-value
Fat	6.89 ± 0.79	6.22 ± 0.64	0.005	7.58 ± 0.69	7.15 ± 0.42	0.022	7.72 ± 0.71	7.31 ± 0.52	0.044
Protein	3.67 ± 0.17	3.29 ± 0.13	0.0001	3.42 ± 0.19	3.14 ± 0.13	0.0001	3.31 ± 0.15	2.97 ± 0.18	0.0001
Lactose	5.33 ± 0.12	5.14 ± 0.11	0.0001	4.96 ± 0.19	4.37 ± 0.15	0.0001	5.31 ± 0.16	4.95 ± 0.14	0.0001
Minerals	0.71 ± 0.03	0.72 ± 0.07	0.560	0.72 ± 0.03	0.73 ± 0.06	0.509	0.74 ± 0.04	0.75 ± 0.06	0.538
pH	6.63 ± 0.09	6.61 ± 0.08	0.462	6.63 ± 0.11	6.60 ± 0.12	0.415	6.64 ± 0.13	6.62 ± 0.06	0.535
Conductivity	4.23 ± 0.06	4.22 ± 0.04	0.538	4.25 ± 0.05	4.24 ± 0.03	0.447	4.24 ± 0.02	4.23 ± 0.05	0.411
SCC (×10^3^)	172.32 ± 5.3	193.18 ± 4.6	0.0001	169.46 ± 6.5	187.59 ± 5.1	0.0001	171.36 ± 4.3	195.48 ± 5.4	0.0001

FMD=Foot-and-mouth disease, SCC=Somatic cell count

**Table-6:** Effect of FMD virus on milk composition of non-pregnant cow and buffalo.

Milk parameters	Cow			Buffalo		
	
	Sero –ve (n = 8)	Sero+ve (n = 8)	p-value	Sero –ve (n = 8)	Sero+ve (n = 8)	p-value
Fat	3.59 ± 0.24	3.15 ± 0.23	0.0001	6.76 ± 0. 19	6.34 ± 0.75	0.020
Protein	3.36 ± 0.22	2.97 ± 0.26	0.0001	3.59 ± 0.19	3.16 ± 0.17	0.0001
Lactose	4.77 ± 0.20	4.41 ± 0.17	0.0001	5.19 ± 0.13	4.85 ± 0.16	0.0001
Minerals	0.67 ± 0.05	0.69 ± 0.08	0.349	0.72 ± 0.05	0.74 ± 0.06	0.259
pH	6.58 ± 0.08	6.53 ± 0.07	0.042	6.61 ± 0.11	6.64 ± 0.08	0.330
Conductivity	4.21 ± 0.06	4.20 ± 0.04	0.538	4.22 ± 0.05	4.21 ± 0.04	0.489
SCC (×10^3^)	151.62 ± 6.3	181.43 ± 5.7	0.0001	168.51 ± 5.7	191.73 ± 4.3	0.0001

FMD=Foot-and-mouth disease, SCC=Somatic cell count

There are many factors affecting the level of reproductive hormones in blood, such as age, species, breed, malnutrition, illness, season, reproductive status, management systems, and nutrition [32]. In this study, the effect of FMDV on estrogen and progesterone levels in non-pregnant and pregnant cattle and buffalo in different stages of pregnancy was studied. FMDV has a significant effect on the elevation of estrogen and reduction of progesterone levels in the blood (Figures-2–5). A nearly identical result was reported by El-Deen *et al*. [33], who found that FMDV significantly reduces progesterone levels in blood. Therefore, we can confidently state that FMDV disturbs various endocrine functions of the reproductive organs, thereby adversely affect cattle and buffalo productivity. Considering the aforementioned results, biosecurity measures should be implemented to prevent the spread of FMDV through raw milk. These measures include careful handling of raw milk during milking, transportation, and storage. Milk samples should be taken regularly from raw market milk to be tested for the presence of FMDV, especially during outbreaks, and only pasteurized milk and dairy products should be consumed.

## Conclusion

The presence of FMDV in 56% and 44% of the tested cattle and buffalos, respectively, indicates the widespread occurrence of FMDV all over Menofia Governorate, Egypt. Milk obtained from infected animals is of poor quality and may cause health risks to consumers. Foot-and-mouth disease virus has a negative effect on milk composition and quality and may disturb some reproductive hormones, which could adversely affect cattle and buffalo productivity. Therefore, accurate diagnosis and effective preventative programs are essential for successful disease control. Milk is an ideal medium for the laboratory diagnosis of FMD and may be particularly appropriate for the surveillance of the disease in dairy herds because it is readily available and easy to collect.

## Authors’ Contributions

AK, RHM, AMZ, and EMB: Prepared conception and design of the study and performed data curation and interpretation. RHM and AMZ: Drafted the manuscript and statistically analyzed the data. AK, RHM, AMZ, and EMB: Collected and analyzed the samples. AK and EMB: Carried out the final writing, critical review, and revision. All authors have read and approved the final manuscript.
